# Nanobody Based Dual Specific CARs

**DOI:** 10.3390/ijms19020403

**Published:** 2018-01-30

**Authors:** Stijn De Munter, Joline Ingels, Glenn Goetgeluk, Sarah Bonte, Melissa Pille, Karin Weening, Tessa Kerre, Hinrich Abken, Bart Vandekerckhove

**Affiliations:** 1 Department of Clinical Chemistry, Microbiology and Immunology, Ghent University, 9000 Ghent, Belgium; stijn.demunter@ugent.be (S.D.M.); joline.ingels@ugent.be (J.I.); glenn.goetgeluk@ugent.be (G.G.); sarahm.bonte@ugent.be (S.B.); melissa.pille@ugent.be (M.P.); karin.weening@ugent.be (K.W.); tessa.kerre@ugent.be (T.K.); 2 Center for Molecular Medicine Cologne (CMMC) and Departement of Internal Medicine, University of Cologne, 50923 Cologne, Germany; hinrich.abken@uk-koeln.de

**Keywords:** CAR T cell, nanobody, antigen escape

## Abstract

Recent clinical trials have shown that adoptive chimeric antigen receptor (CAR) T cell therapy is a very potent and possibly curative option in the treatment of B cell leukemias and lymphomas. However, targeting a single antigen may not be sufficient, and relapse due to the emergence of antigen negative leukemic cells may occur. A potential strategy to counter the outgrowth of antigen escape variants is to broaden the specificity of the CAR by incorporation of multiple antigen recognition domains in tandem. As a proof of concept, we here describe a bispecific CAR in which the single chain variable fragment (scFv) is replaced by a tandem of two single-antibody domains or nanobodies (nanoCAR). High membrane nanoCAR expression levels are observed in retrovirally transduced T cells. NanoCARs specific for CD20 and HER2 induce T cell activation, cytokine production and tumor lysis upon incubation with transgenic Jurkat cells expressing either antigen or both antigens simultaneously. The use of nanobody technology allows for the production of compact CARs with dual specificity and predefined affinity.

## 1. Introduction

Adoptive chimeric antigen receptor (CAR) T cell therapy seems to be remarkably effective for acute lymphoblastic leukemia (ALL). CD19 CAR T cells eradicate late stage leukemia in 70–90% of treated patients. The main cause of failure of CAR T cell therapy is antigen escape, yielding CD19 negative leukemic cells that are no longer susceptible to CD19 CAR T cells. Up to 30% of relapsed ALL patients have been reported to be due to loss or downregulation of the CD19 epitope. CD19 antigen escape can arise by different mechanisms, including differential splicing, missense mutations, or lineage switch [1,2,3,4,5,6,7,8]. Antigen escape is not unique to CAR therapy and has been reported for other targeted therapies, such as serine/threonine-protein kinase B-raf (BRAF) inhibitors and PD1 blockade [9,10,11]. These observations highlight the universal potential of tumor cells to undergo tumor editing during targeted therapies. 

Targeting two or more antigens simultaneously could be an option to reduce outgrowth of antigen escape variants causing relapse and treatment failure. This has been shown earlier in the context of targeted immunotoxin treatments [12,13,14]. Different CAR approaches have been tested to achieve bispecificity by (i) mixing two T cell lines, each expressing one CAR specific for one antigen (mixing); (ii) transducing T cells to simultaneously express two different CARs (dual signaling CAR, combining) or (iii) transducing T cells to express one single CAR that consists of two antigen-binding domains in tandem (TanCAR, multiplexing). Each approach has its advantages and shortcomings. It has been shown that mixing different CAR T cell populations may result in the preferential outgrowth of just one CAR T cell population. The dual signaling CAR approach is severely compromised by the limited packaging size of viral vectors [15,16,17]. On the other hand, multiplexing of CARs has been successful for the combination of CD19 and HER2/neu and for CD20 and CD19 [17,18]. Other combinations have been published [19]. The bispecific CARs or TanCARs were able to recognize antigens in a Boolean OR-gate fashion: either antigen was sufficient to induce robust T cell activation, expansion and function. When both antigens were presented, a synergistic effect was observed [17,18]. However, the generation of TanCARs is challenging due to the potential cross-pairing between the variable light (VL) and variable heavy (VH) chains of different scFvs and the variable loss of affinity that may occur in the design of the scFvs [15,20]. In addition, there is also a restraint on the size due to the limited packaging capacity of retroviral vectors [21,22]. 

As an alternative to scFvs, nanobodies can be used. Nanobodies consist of the VH domain of heavy chain only antibodies of Camilidae. The heavy chain only antibodies were first described by Hamers-Castermans [23]. Further characterization revealed the special structure of these antibodies; they are composed of two heavy chains in which the CH1 domain is lacking. Due to the absence of the CH1 domain, the antibodies do not have a light chain. As a result, the antigen-binding capacity is confined to only one variable domain and not two. These single antigen-binding domains can be cloned, easily expressed and retain the affinity for the specific antigen [24,25]. Their strict monomeric behavior and their small size makes them ideal building blocks for multidomain constructs. Nanobodies do not interact with each other. Recent clinical trials have shown possible success with multidomain nanobody based drugs [26,27]. The use of mouse based scFvs can result in human anti-mouse antibodies. This immunogenicity can lead to adverse events and loss of efficacy during CAR therapy [28,29]. Nanobodies, on the other hand, are only weakly immunogenic, due to extensive sequence identity with the human VH gene family III [24,25]. In addition, Vincke et al. have developed a humanized scaffold nanobody onto which the antigen-binding loops of specific nanobodies can be grafted [30]. Clinical trials have shown virtually absent anti-nanobody antibody induction [26,27]. 

Long lasting remission seems to be dependent on long term persistence of CAR T cells. Long term survival of T cells depends on the exhaustion profile of the infused T cells [7,31]. Long et al. have shown that CAR aggregation on the cell membrane may result from interactions within the scFv framework and induces CAR CD3ζ domain phosphorylation, tonic T cell activation and ultimately T cell exhaustion [31]. It was described earlier that scFvs have a strong tendency to self-aggregate [32]. Nanobodies could offer a solution to this issue as nanobodies are single domain antigen-binding moieties hat do not interact with one another. These characteristics makes nanobodies ideal building blocks for CARs. Monospecific nanobody based CARs have been generated earlier and showed similar functionality as scFv-based CARs [33,34,35,36]. 

As a proof of principle, we generated and validated a bispecific CAR, based on nanobodies (nanoCAR). The variable domains of the scFv were replaced by two nanobody VHs, each specific for a different antigen: CD20 and HER2. T cells expressing the bispecific nanoCAR were able to kill tumor cells expressing CD20, HER2 or both. The affinity of the nanoCAR for the antigen was similar to the original nanobodies, allowing the straightforward design of a bispecific CAR with predetermined affinities. 

## 2. Results and Discussion

**Figure 1:**
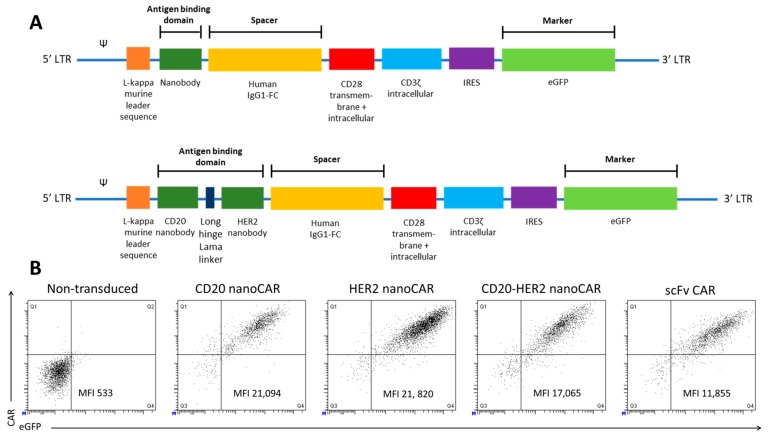
Nano chimeric antigen receptorS (CARs) are stably expressed on T cells at levels comparable to an scFv based CAR. (**A**) Scheme of the second generation nanoCAR constructs used to retrovirally transduce T cells. The antigen-binding domain is linked to the constant regions of the Fc tail of the human IgG1 antibody heavy chain, which is linked to the transmembrane and intracellular CD28 and intracellular CD3ζ chain. (**B**) Human peripheral blood T cells were activated and retrovirally transduced to express a nanoCAR. CAR expression was measured by flowcytometry on eGFP^+^ sorted cells after staining with a phycoerythrin (PE)-conjugated human anti-IgG antibody, which binds the spacer domain in the CAR. Non-transduced and scFv-CAR transduced T cells were used as controls. The figure is representative of five different healthy donors.

We first validated the CD20 and HER2 monomeric nanoCARs. The coding sequence for the specific nanobody was inserted in the retroviral plasmid encoding a CAR consisting of the human IgG1 CH2CH3 (Fc) spacer, the CD28 transmembrane domain, the CD28 and the CD3ζ intracellular signalling domain (Figure 1A). We generated a bispecific nanoCAR subsequent to validation of the monomeric nanoCAR. The nanobody targeting CD20 was linked through the structural upper hinge of the lama IgG2a with the HER2 specific nanobody (Figure 1A). Peripheral blood derived CD4^+^ and CD8^+^ T cells were subsequently transduced to express a CD20 nanoCAR, a HER2 nanoCAR and the bispecific nanoCAR. T cells expressing the CARs were sorted and expanded. NanoCARs were expressed at a similar or higher level, compared to an scFv-based CAR (Figure 1B). More importantly, the bispecific nanoCAR was stably expressed at high levels.

**Figure 2:**
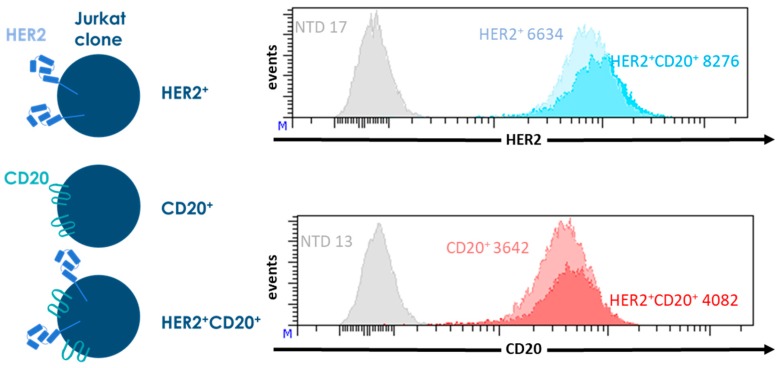
Characterization of the transgenic Jurkat lines expressing CD20, HER2, or both, that are used as target cells in the experiments shown in Figure 3. Jurkat cells were retrovirally transduced with the sequences coding for CD20 of for HER2 truncated at position 695 (HER2Δ695). The transgenic Jurkat cells were stained for CD20 and HER2 expression and analyzed by flowcytometry. As antigen negative control, non-transduced Jurkat cells (NTD) were used, MFIs are shown for each Jurkat clone.

To accurately determine the activity of the CAR T cells, we generated Jurkat lines expressing CD20 (CD20^+^), HER2 (HER2^+^) or both (CD20^+^HER2^+^). These Jurkat lines were used as targets in all of our experiments. Wild type Jurkat cells do not express CD20 or HER2. We generated three stable transgenic Jurkat clones expressing CD20, HER2, or both, at similar levels. In this way, we could exclude a possible effect of antigen density on CAR function. Non-transduced Jurkat cells were used as an antigen negative control (Figure 2). 

**Figure 3:**
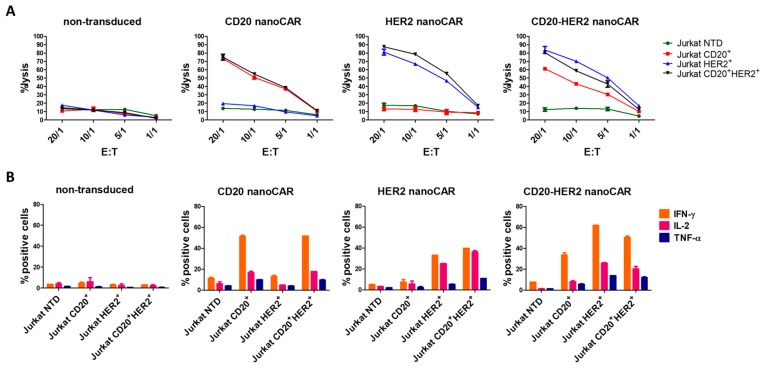
Validation of the monospecific and tandem nanoCARs. (**A**) Cell lysis of Jurkat target cells expressing CD20, HER2, or both, after 4 h co-incubation with T cells expressing the monospecific or tandem nanoCAR in different effector-target ratios. Reported values are means of duplicate determinations with error bars indicating the standard deviation. Results are representative of two independent experiments, performed with three different donors. (**B**) Cytokine production of nanoCAR transduced T cells was analysed by intracellular staining after co-incubation with Jurkat cells. Mean percentages of interferon-γ (IFN-γ), interleukin-2 (IL-2) and tumor necrosis factor-α (TNF-α) positive cells are shown. Error bars represent standard deviations. The data is representative of two independent experiments, performed with three different donors.

We first evaluated the cytotoxic activity of nanoCAR transduced T cells in a standard 4 h chromium-51 release assay (Figure 3). Non-transduced T cells were not able to lyse antigen positive cell lines. We saw robust killing of antigen positive cells by the monospecific and bispecific nanoCAR T cells. The cytotoxic activity was nanoCAR and antigen related since antigen negative Jurkat cells and non-transduced T cells did not elicit cell lysis. While the tandem nanoCAR T cells killed HER2 postive Jurkat cells in the same fashion as the HER2 nanoCAR T cells, we saw a lower killing efficiency by the tandem nanoCAR T cells for single CD20 positive Jurkat cells. However, the killing efficiency was restored when both HER2 and CD20 were present on the Jurkat cells (Figure 3A). 

Next, we examined the cytokine production of transduced and non-transduced T cells after challenging them with the different Jurkat lines. Jurkat cells were co-incubated for 16 hr with T cells. Subsequently, the T cells were labelled for intracellular interferon-γ (IFN-γ), interleukin-2 (IL-2) and tumor necrosis factor α (TNF-α). The transduced T cells were able to produce IFN-γ, IL-2 and TNF-α. Both single antigen expressing Jurkat cell lines were capable of stimulating mono- and bispecific T cells. The tandem nanoCAR T cells showed a strong response when stimulated with HER2positive Jurkat cells but the response on single CD20 positive cells was lower than the response of the monospecific nanoCAR T cells. This observation was in line with the cytotoxicity experiments (Figure 3B).

Both the cytokine secretion and cytotoxicity assay clearly showed that the bivalent nanobody based CAR acts in a Boolean OR-gate fashion. However, we observed no additive or synergistic effects. Furthermore, the functionality of the tandem nanoCAR T cells was lower, compared to the CD20 mono-specific nanoCAR T cells, when these were stimulated with CD20 single positive Jurkat cells. 

**Figure 4:**
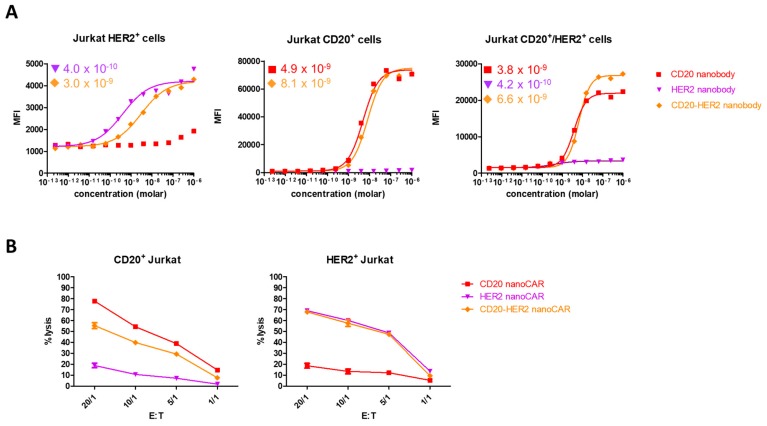
It is not the affinity of the different CAR structures that affects their function, but rather, the CAR structure. (**A**) Affinity determination of the monospecific of bispecific nanobody constructs. Affinities are shown in molar concentration. (**B**) Lysis by mono-specific or tandem nanoCAR T cells against Jurkat target cells expressing one antigen. Data show the mean values of duplicates and are representative of three independent experiments on three different donors.

We wondered whether the absence of an additive effect could be the result of impaired simultaneous binding of CD20 and HER2 by the tandem nanoCAR. We generated the respective monospecific and bispecific nanobodies and tested these in a flowcytometric binding assay. While the CD20 nanobody in the tandem CAR had a higher binding affinity for CD20 (4.9 × 10^−9^ M for the monospecific and 8.1 × 10^−9^ M for the bispecific nanobody), the affinity of the HER2 nanobody domain dropped from 4.0 × 10^−10^ M to 3.0 × 10^−9^ M. When the nanobody constructs were tested for binding with both antigens simultaneously, we did not detect an additive effect (Figure 4A). While the affinity for HER2 dropped, we observed no difference in killing potential of the tandem nanoCAR for single HER2 positive Jurkat cells, compared with the lytic activity of the HER2 nanoCAR transduced T cells. However, while the affinity for CD20 increased when targeted by our tandem nanoCAR, we saw a decrease in lytic potential when tested against a single CD20 positive Jurkat cell line, compared with the monospecific CD20 nanoCAR (Figure 3A and Figure 4B). In conclusion, we did not find a correlation between affinity and functionality. 

We have shown for the first time that it is possible to generate a bispecific CAR based on nanobodies. This CAR act as a typical Boolean OR-gate: one of the two antigens is sufficient to elicit robust T cell activation, cytokine production and cytotoxic activity. 

However, some optimization remains necessary. The long hinge lama linker that is used here to connect both nanobodies is bulkier and less flexible than the standard glycine-serine hinges (Figure 1). However, the linker is flexible enough to allow both nanobodies to move freely [37]. As a result, it is unlikely that one nanobody interferes with the binding of the other to its antigen. This is in line with the affinity determinations in which we observed binding of either CD20 or HER2, although an additive effect was not found. Furthermore, the nanoCAR T cells stimulated with HER2 positive target cells elicited a stronger T cell response than when stimulated with CD20 positive cells, despite similar binding affinity of the tandem nanoCAR for CD20 and HER2. This could be due to a suboptimal distance between the cell membranes of the CAR T effector cells and target cells at the immunological synapse, induced by the binding of the tandem nanoCAR to CD20 and HER2. Different groups have shown that the distance between the CAR T cell and their target cell is critical for T cell functionality. This distance is determined by the length of the spacer domain and the epitope location targeted by the antigen binding domain. If a monospecific CAR recognizes an epitope located close to the cell membrane of the target cell, then T cell activation is optimal when the CAR is designed with a long spacer domain. In contrast, when the targeted epitope is located more distally from the cell membrane, a short spacer seems to be optimal for T cell function [38,39,40,41]. This can be explained by the kinetic segregation model. According to this model, the immunological synapse, formed by the interaction of a T cell with the target cell has to generate close contact zones, able to physically exclude the large inhibitory CD45 or CD148 phosphatases. If these close contact zones do not form, inhibitory phosphatases enter the synapse and abort T cell activation [42,43,44]. We used a long spacer in the tandem nanoCAR. The distance from the HER2 nanobody domain to the cell membrane of the T cells is the length of the spacer, while the distance from the CD20 nanobody domain to the cell membrane is the combined length of the spacer, the HER2 nanobody and the linker between the HER2 and CD20 nanobody. The long distance from the CD20 nanobody to the cell membrane could result in an immunological synapse that is too large. Therefore, inhibitory phosphatases may enter the synapse and disturb T cell activation. 

One shortcoming of our experiments is the sole use of in vitro functional tests. These assays do not always predict in vivo effectiveness of multiple antigen targeted therapeutics. Therefore, in vivo experiments should be carried out [14]. 

This tandem CD20 HER2 nanoCAR was designed as proof of principle CAR. Tandem nanoCARs for therapeutic purposes should be directed to relevant targets, such as CD19, CD20 and/or CD22 for the treatment of B cell leukemias or CLL-1, CD33 and/or CD123 for the treatment of acute myeloid leukemia. For the design of these tandem nanoCARs, one should consider the structure of the targeted proteins and the CAR architecture. For instance, CD20 is a multipass transmembrane protein with no protruding extracellular domains [45], while CD19, a single pass membrane protein and member of the Ig like protein family, is a typical transmembrane protein, consisting of a large extracellular domain [46]. Construction of a tandem nanoCAR should take into account spacer length, epitope location, linker length and position of the nanobodies in the CAR construct (Figure 1). Since CD20 is a multipass protein, the epitope of the generated nanobody will be close to the cell membrane of the leukemic cell, while the epitope of a CD19 specific nanobody may be more distal to the leukemic cell membrane. Therefore, the CD20 nanobody domain should be at the N-terminal side of the CAR. The CD19 nanobody domain, on the other hand, should be close to the cell membrane of the T cell and therefore connected to the cell membrane with a short spacer domain, such as a CD8α-hinge or an IgG4-hinge region. The linker length between the CD20 and CD19 nanobodies should be determined empirically. All these variables should be tested in vitro and eventually, in vivo.

In conclusion, we have shown that bivalent nanobody based CARs are easily designed, expressed and are functional. An optimally designed nanoCAR may be advantageous in the treatment of leukemias as it should be able to prevent antigen escape.

## 3. Materials and Methods 

### 3.1. Culture of Cell Lines

All the Jurkat lines were cultured, as per American Type Culture Collection (ATCC, Manassas, Virginia) recommendations, in standard complete medium, consisting of IMDM (Gibco, Invitrogen, Merelbeke, Belgium), supplemented with 10% fetal calf serum (FCS, Gibco, Invitrogen), 2 mM L-glutamine (Gibco, Invitrogen), 100 IU/mL penicillin (Gibco, Invitrogen) and 100 IU/mL streptomycin (Gibco, Invitrogen) (complete IMDM, cIMDM). The JY cell line, a human HLA-A2^+^ Eppstein Barr virus (EBV)-immortalized B-cell line, was obtained from the ATCC and cultured in cIMDM. 

### 3.2. Production of Retroviral Vectors

The different constructs, as shown in Figure 1A, were generated by cloning a gBlock (IDT) coding for the murine kappa leader and the antigen-binding domain into the LZRS-IRES-eGFP vector that already contained a second generation CAR, using BamHI [47]. Viral particles were produced using the Phoenix packaging cell line. Retroviral supernatant was collected at day 14 after transfection and puromycin selection and frozen until use. 

### 3.3. Generation of NanoCAR Expressing Human T Cells

Buffy coats from healthy donors were obtained from the Belgian Red Cross and used following the guidelines of the Medical Ethical Committee of Ghent University Hospital (CG20171208A, 8 December 2017), after informed consent had been obtained, in accordance with the Declaration of Helsinki. Mononuclear cells were isolated by Lymphoprep (Axis-shield, Dundee, UK) gradient centrifugation. T cells were enriched by magnetic activated cell sorting using Streptavidin microbeads (MACS beads, Milteyni, Leiden, The Netherlands), after staining with homemade CD4-biotin (OKT4) and CD8-biotin (OKT8). T cells were stimulated with CD3/CD28 T-cell activation Dynabeads (Life Technologies, Merelbeke, Belgium) at a 1:1 bead:cell ratio, in cIMDM medium, in the presence of 10 ng/mL IL-12, and retrovirally transduced on retronectin coated plates (TaKaRa, Saint-Germain-en-Laye, France) 72 h after stimulation. Cells resuspended in retroviral supernatant were centrifuged for 90 min at 2300 rpm at 32 °C. Dynabeads were removed before transduction. 

Transduced cells were detected by eGFP expression or by an anti-IgG antibody directed against the human IgG1 spacer domain present in the extracellular domain of both CARs. Transduced cells were sorted and expanded on irradiated allogenic feeder cells, consisting of a mixture of 40-Gy irradiated peripheral blood mononuclear cells and 50-Gy irradiated JY cells. Cells were cultured in cIMDM (Gibco, Merelbeke, Belgium), supplemented with 1 μg/mL phytohemagglutinin (PHA, Sigma–Aldrich, Diegem, Belgium). IL-2 (40 IU/mL) (Roche, Vilvoorde, Belgium) was added on day 5 and day 10. Cells were restimulated every 7–14 days. 

### 3.4. Flow Cytometry and Antibodies

Staining of surface markers was performed in DPBS with 1% FCS using the antibody concentration recommended by the supplier. Intracellular staining was performed following the supplier’s protocol using BD Cytofix&Cytoperm (BD Biosciences, Erebodegem, Belgium). Flow cytometric analysis was performed on the LSR II and cell sorting on the ARIA II (both BD Biosciences). All populations analyzed were devoid of dead cells based on propidium iodide negativity and of doublets based on FSC-A FSC-W ratios. The following anti-human monoclonal antibodies were used: PE-conjugated—IgG-Fc (eBioscience, Merelbeke, Belgium); allophycocyanin (APC)-conjugated—CD20 (BD Biosciences), HER2 (BD Biosciences), CD8α (BD Biosciences), CD4 (BD Biosciences); APC-Cy7-conjugated—CD8α (BD Biosciences); biotine-conjugated—CD4 (OKT4, homemade), CD8 (OKT8, homemade). 

### 3.5. ^51^Chromium Release Assay

Target cells were labelled with ^51^Chromium (Perkin Elmer, Zaventem, Belgium) for 90 min at 37 °C, washed and added at 10^3^ cells per well to various ratios of effector T cells in 96 well V-bottomed plates (NUNC, Thermo Fisher Scientific, Merelbeke, Belgium). After 4 h of co-incubation, the supernatant was harvested and measured in a 1450 LSC & Luminescence Counter (Perkin Elmer, Zaventem, Belgium). Specific lysis was calculated, as follows: (experimental release–spontaneous release)/(maximal release–spontaneous release) × 100%. 

### 3.6. Flowcytometric Determination of Cytokine Production

Two hundred thousand feeder culture expanded T cells were stimulated by co-incubation with Jurkat lines expressing the relevant antigen at 10^5^ cells in 96-well U-bottom plates. After 1 h of stimulation, BD GolgiPlug (BD Biosciences) was added and after an additional 16 h of stimulation, the cells were harvested, permeabilized, labelled and analyzed by flowcytometry for cytokine expression using TNF-α-PE-Cy7, IFN-γ-PE and IL-2-APC (all three from BD Biosciences). 

### 3.7. Affinity Determination

Jurkat lines expressing CD20, HER2 or both were coated in 96-well U-bottom plates (NUNC, Thermo Fisher Scientific) and nanobody was added in different dilutions. After incubation for 30 min at 4 °C, cells were labelled with mouse anti-FLAG antibody (Sigma-Aldrich) for 30 min at 4 °C, washed and labelled with goat anti-mouse-APC (Jackson ImmunoResearch Laboratories, St-Martens-Latem, Belgium) for 30 min at 4 °C. Cells were analyzed by flowcytometry after a final wash step.
